# The Occipitalis Muscle as an Adjunct Superficial Landmark for the Transverse Sinus and Transverse-Sigmoid Junction: An Anatomical Study With Application to Posterior Cranial Fossa Surgery

**DOI:** 10.7759/cureus.39723

**Published:** 2023-05-30

**Authors:** Aishwarya Gilkes, Mathangi Rajaram-Gilkes, Juan J Cardona, Francisco Reina, Ana Carrera, Joe Iwanaga, Aaron S Dumont, Carmine Antonio Donofrio, Filippo Badaloni, Antonio Fioravanti, R. Shane Tubbs

**Affiliations:** 1 Department of Anatomical Sciences, St. George’s University, St. George’s, GRD; 2 Department of Medical Education, Geisinger Commonwealth School of Medicine, Scranton, USA; 3 Department of Neurosurgery, Tulane Center for Clinical Neurosciences, Tulane University School of Medicine, New Orleans, USA; 4 Department of Medical Sciences, Clinical Anatomy, Embryology and Neurosciences Research Group (NEOMA) Faculty of Medicine, University of Girona, Girona, ESP; 5 Department of Neurology, Tulane University School of Medicine, New Orleans, USA; 6 Department of Structural and Cellular Biology, Tulane University School of Medicine, New Orleans, USA; 7 Department of Oral and Maxillofacial Anatomy, Graduate School of Medical and Dental Sciences, Tokyo Medical and Dental University, Tokyo, JPN; 8 Dental and Oral Medical Center, Kurume University School of Medicine, Kurume, JPN; 9 Department of Molecular and Translational Medicine, Division of Biology and Genetics, Faculty of Medicine, University of Brescia, Brescia, ITA; 10 Department of Neurosurgery, ASST Cremona, Cremona, ITA; 11 Department of Neurosurgery, Istituto di Ricovero e Cura a Carattere Scientifico (IRCCS) Istituto delle Scienze Neurologiche di Bologna, Bologna, ITA; 12 Department of Surgery, Tulane University School of Medicine, New Orleans, USA; 13 Department of Neurosurgery, Ochsner Neuroscience Institute, Ochsner Health System, New Orleans, USA

**Keywords:** landmark, transverse sigmoid sinus junction, transverse sinus, occipitalis muscle, neurosurgery, anatomy

## Abstract

Introduction: Although neuronavigation systems are widely used for identifying deep intracranial structures, additional superficial anatomical landmarks can be useful when this technology is not available or is not working properly. Herein, we investigate the potential of the occipitalis muscle (OM), rarely mentioned in neurosurgical literature, as a superficial landmark for the transverse sinus (TS) and transverse-sigmoid sinus junction (TSJ).

Methods: Eighteen adult cadaveric heads underwent dissection. The borders of the OM were identified and measured. The muscle was then removed and the bone underlying the muscle was drilled. The relationships between the OM and the underlying dural venous sinuses were then investigated by using a surgical microscope.

Results: The OM is a quadrangular-shaped muscle, that invariably crosses the lambdoid suture, showing relationships with the TS inferiorly and the TSJ laterally. The medial border was located a mean of 2.7 cm from the midline and its lower edge was a mean of 1.6 cm above the TS. The inferior border was found between the lambdoid suture and the superior nuchal line in all the specimens. The medial half of the inferior margin was placed on average 1.1 cm superiorly to the TS while the lateral margin ran just above or over the TS. The lateral border was located a mean of 1.1 cm medially to the asterion and approximated the mastoid notch, being within 1-2 cm from it. The TSJ was between 2.1 and 3.4 cm lateral to OM lateral border.

Conclusion: A combination of superficial anatomical landmarks can be useful for surgical planning. We found that the OM represents a valuable aide for neurosurgeons and is a reliable landmark for the deeper-lying TS and TSJ.

## Introduction

The occipitalis muscle (OM) is a very thin quadrangular-shaped muscle located on the posterolateral surface of the cranium, posteromedially to the pinnae. It originates from the mastoid process of the temporal bone and the lateral two-thirds of the superior nuchal line, usually sitting in the area just above the supreme nuchal line. It inserts into the galea aponeurotica anteriorly.

Despite a minimal function in humans, when paired with the frontalis muscle, which also attaches to the galea aponeurotica, the OM can move the scalp. The tension created by the OM better allows the frontalis muscle to create facial expressions such as surprise or horror, that require elevation of the skin of the forehead. The OM receives its motor innervation from the posterior auricular branch of the facial nerve and its blood supply and drainage come from the occipital artery and vein, respectively.

Several studies have examined various superficial cranial landmarks for identifying deeper-lying dural venous sinuses [1-3]. In this study, we hypothesized that the OM might be a reliable superficial landmark for identifying the deeper-lying transverse sinus (TS) and transverse-sigmoid sinus junction (TSJ).

## Materials and methods

Five fresh frozen and four formalin (18 sides) fixed human adult cadaveric heads were dissected. Four specimens were also latex injected. Five male and four female specimens were included in the study, with an age at death that ranged between 44 and 89 years (mean 69.5 years). No specimen had a known history of surgery, trauma, or pathologies involving the dissected regions. The skin and superficial fascia overlying the OM and upper craniocervical musculature were removed. The borders of the OM were identified and measured using a microcaliper (Mitutoyo, Japan). A surgical skin marker was used to outline the muscle’s borders. The muscle was then removed and the bone underlying the muscle was drilled (Stryker, Kalamazoo, MI, USA). The relationships between the OM and the underlying dural venous sinuses were then assessed using a surgical microscope (Zeiss, Germany). Selected specimens underwent sagittal sections through the head for identification of the overlying OM and relationships to the underlying dural venous sinuses.

Statistical analysis was performed with Jeffrey's Amazing Statistics Program (JASP) (16.4, Amsterdam, The Netherlands). A two-tailed probability (p) value ≤0.05 was considered statistically significant. Tulane University Institutional Review Board does not require approval of non-patient/living human studies. Thus, as our study used cadavers, approval was not required. The authors state that every effort was made to follow all local and international ethical guidelines and laws that pertain to the use of human cadaveric donors in anatomical research [4].

## Results

**Figure 1:**
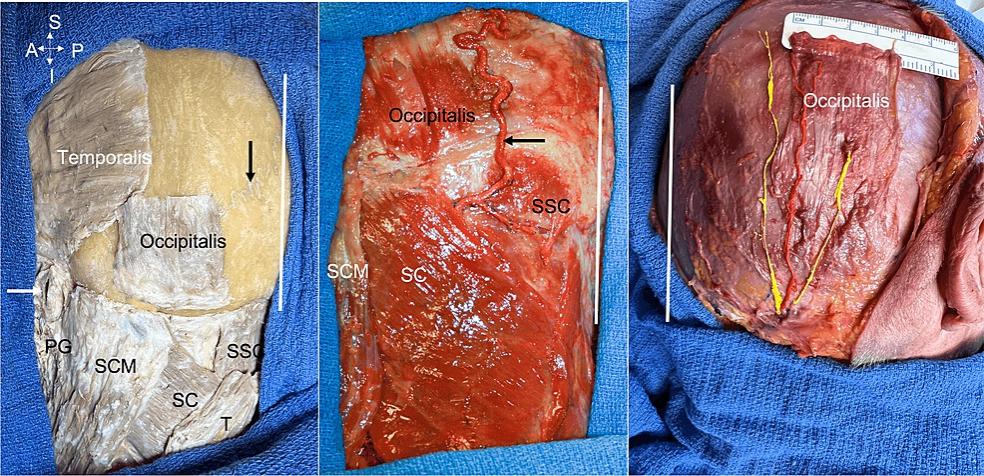
Three dissections of the occipitalis muscle. Left: The muscle borders of the left occipitalis are defined and its relationships to surrounding structures are shown; lambdoid suture (black arrow), external acoustic meatus (white arrow), parotid gland (PG), sternocleidomastoid (SCM), splenius capitis (SC), semispinalis capitis (SSC), trapezius (T). Middle: Left occipitalis muscle. Note the semispinalis capitis (SSC) and occipital artery (arrow). Right: Right occipitalis muscle. Note the relationships between the occipitalis muscle and the overlying occipital artery and the greater occipital nerve. For each image, the vertical white line marks the midline.

The OM was found in the galeal layer, superficial to the superficial layer of the temporalis fascia on all sides. The auricularis muscles were just anterior to the OM. Six OMs were quadrangular and 12 were rectangular in shape. The muscle fibers traveled in a more or less vertical direction. Branches of the occipital artery and greater occipital nerve ran superficially to the muscle (Figure 1).

**Figure 2:**
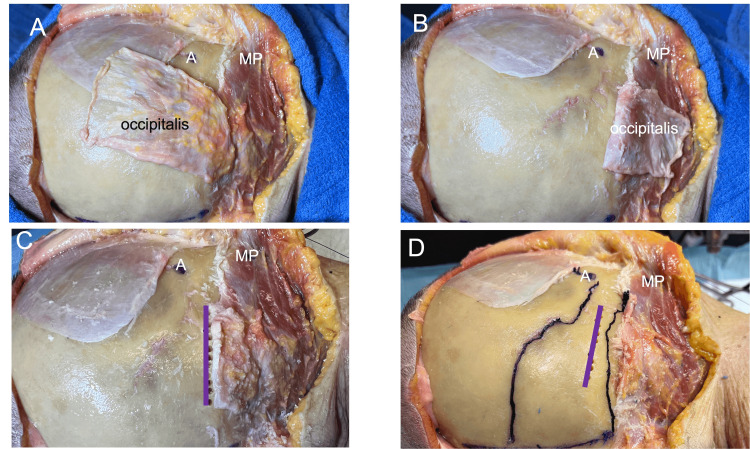
In the lateral position, the stepwise dissection of the occipitalis muscle. A. Note the muscle is intact the asterion (A) anteriorly positioned and the mastoid process (MP) seen inferolateral. B. The occipitalis is reflected while maintaining its inferiorly attachment. C. The inferior attachment of the muscle (purple line) has pilot holes drilled into the skull to identify this line’s relationship to the underlying transverse sinus. D. The relationships between the inferior attachment of the occipitalis (straight purple line) and the lambdoid suture and superior nuchal line, the left and right of it, respectively. The midline is shown at the very bottom of the image as the horizontal purple line.

The mean width (horizontal distance), length (vertical distance), and thickness of the OM were 45 cm (range: 40-56 cm), 59 cm (range: 55-76 cm), and 0.27 mm (range: 0.21-0.35 mm), respectively. The OM invariably crossed the lambdoid suture. The medial border of the muscles was located on average 2.7 cm (range: 2.3-3.5 cm) from the midline. The lower edge of the medial border was 1.6 cm in the mean (range: 1.1-2.2 cm) superior to the TS. The inferior border of the OM was found between the lambdoid suture and the superior nuchal line on all sides (Figure 2), inserting on or near the supreme nuchal line, which was present on three out of nine specimens, all of which were males.

**Figure 3:**
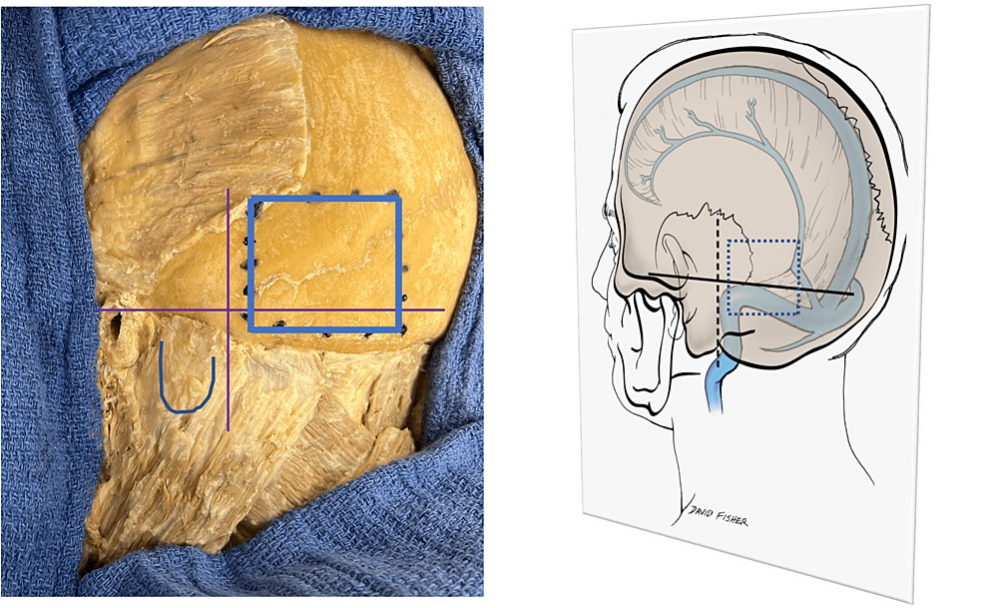
Left: Posterolateral view of the left skull with the outline of the occipitalis (blue box). The mastoid is outlined as well as the mastoid notch (vertical line) and the upper border of the zygomatic arch/inion line (horizontal line). Right: Schematic drawing of the left image with the underlying dural venous sinuses shown in blue. Note that the inferior border of the occipitalis (dotted box) overlies the transverse sinus and that its lateral edge is over the transverse sigmoid junction.

**Figure 4:**
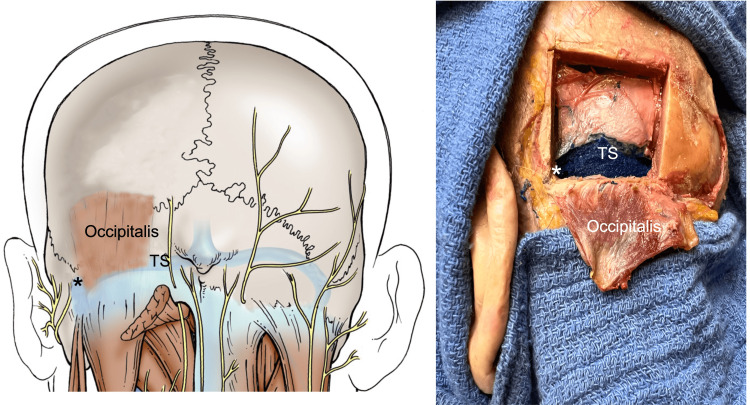
Left: Schematic drawing of the posterior occiput. On the left side, note the position of the occipitalis muscle and its superficial relationship inferiorly to the transverse sinus and TSJ (*). As on the left image, the mastoid notch (vertical dotted line) and zygomatic arch/inion line (horizontal line) are shown. Right: Left cadaveric dissection where the occipitalis has been reflected inferiorly and its borders marked with the underlying skull removed. Note the relationship between the lower margin of the occipitalis attachment and the underlying transverse sinus (TS). The * marks the transverse sigmoid junction.

**Figure 5:**
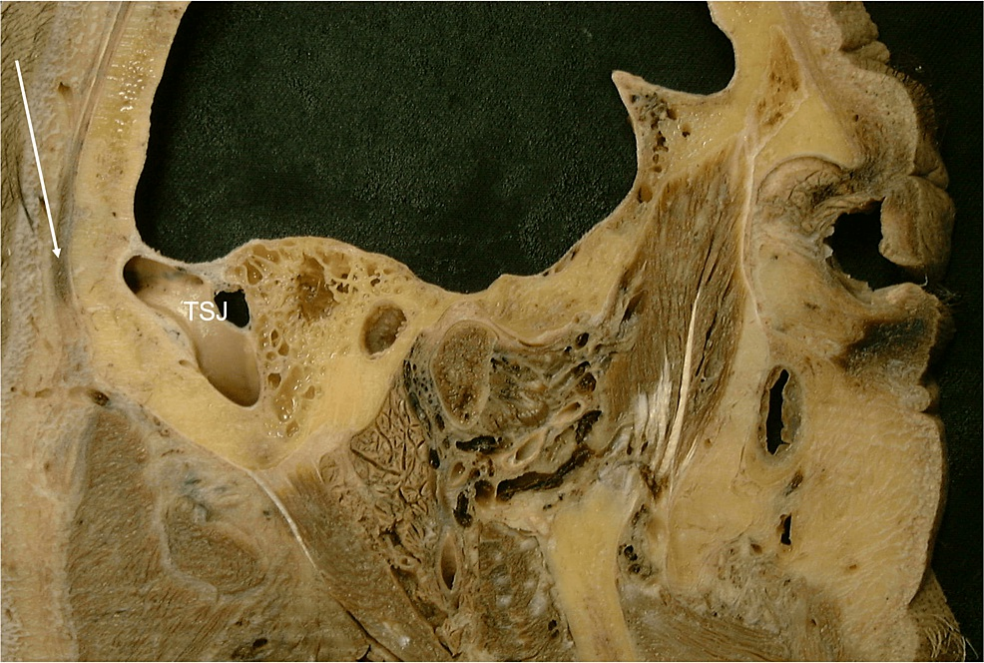
Sagittal section through the head noting the relationship with the lateral edge of the occipitalis muscle (arrow) and the underlying transverse-sigmoid junction (TSJ).

The medial half of the inferior margin was placed on average 1.1 cm superiorly to the TS (range 0.7-1.3 cm), while the lateral one ran just above or over the TS. The lateral border was located at a mean of 1.1 cm (range: 0.7-1.3 cm) medially to the asterion (Figure 2) and approximated the mastoid notch on all sides, being distant within 1-2 cm (mean: 1.2 cm) from this landmark (Figure 3). The TS was on average 1.1 cm (range: 0-1.5 cm) superior to the inferior border of the OM (Figure 4) and the TSJ was on average 2.5 cm (range: 2.1-3.4 cm) lateral to the muscle’s lateral border (Figure 5).

Although the inferior border of the OM tended to be closer to the TS and TSJ on the right sides, the difference between the sides did not reach statistical significance. The absence of the supreme nuchal line in females was significantly different compared with males (p<0.0001). However, no other statistically significant differences were found between males and females.

## Discussion

We found that the lower border of the OM was predictably related to the TS and laterally, the TSJ. Therefore, such an anatomical landmark can be useful as an adjunct to other superficial landmarks e.g., asterion in localizing these deeper-lying structures.

Comparative anatomy

The OM differs in structure among different primates. As reported by Rui et al. [5], the Lemur, Propithecus, Loris and Nycticebus, and Tarsius all have OMs. However, in the Propithecus, Loris, and Nycticebus, the OM is made of the occipitalis proprius and a ‘cervical-auriculo-occipitalis’ muscle. In the Porpithecus and the Loris, the ‘cervical-auriculo-occipitalis’ muscle passes from the occipital region to the external ear. In its route, it passes laterally to the occipitalis proprius which runs from the occipital region to the galea aponeurotica, as seen in humans.

Clinical implications

As a superficial muscle, the OM can be easily stimulated using electrodes. Yildirim et al. [6] used repetitive nerve stimulation of the OM for the diagnosis of myasthenia gravis. By extension of this knowledge, the OM can be used in addition to the nasalis muscle in the electrodiagnosis of myasthenia gravis [7]. Furthermore, the OM has shown some use in assessing the prognosis of peripheral facial palsy. Uzun et al. [8] used electroneurography to assess peripheral facial palsy and found that the OM was better able to reflect the extent of damage and fiber degeneration, earlier than the standard use of the nasalis muscle.

Interventional considerations

The OM has also been studied in patients with occipital neuralgia, treated with botulinum toxin injections [9]. In some patients with occipital neuralgia, trigger points in the suboccipital region can refer pain to the OM and temporalis muscles [10].

Many studies have evaluated the use of different surface landmarks to locate the TSJ. In the study conducted by Hall and Peter [11], 10 different anatomical landmark methods for locating the TSJ were analyzed. The study found that the mean distance to this location showed two tiers of accuracy: <10mm from the keyhole, and >10 mm from the keyhole. The methods proposed by Li et al. [12], Ribas et al. [13], Tubbs et al. [14], Teranishi et al. [15], and Day et al. [16] showed more accurate estimates of the TSJ (<10 mm from the keyhole) while those proposed by Lang and Samii [17], Rhoton [18], Avci et al. [19] were less precise estimates of the TSJ (>10 mm from the keyhole) [11]. The asterion, which previously was thought to overlie the TSJ, was found to have a mean distance of >13 mm from the keyhole [11].

Kubo et al. [20] have proposed the use of the digastric groove as a surface landmark to avoid the TSJ while determining the keyhole position in the lateral suboccipital approach. They have found that when the mastoid notch line (extension of the digastric groove) is used as the x-axis, and the digastric groove point (posterior edge of the digastric groove and vertical line from this point) is used as the y-axis, the TSJ was superior to the groove line. They concluded that the optimal keyhole position for avoiding the TSJ was 20 mm from the groove point, and subjacent to the groove line.

In the past, other muscles of the occipital temporal regions have been used as superficial anatomical landmarks for the underlying dural venous sinuses. For example, the attachment of the longissimus capitis onto the mastoid process has been found to attach on or very near the sigmoid sinus [21]. Such superficial muscles as this or the OM as shown in the current study can be additional information for neurosurgeons and serve to confirm imaging studies or intraoperative navigation. As bony landmarks are sometimes difficult to see during surgery, such as the asterion, especially in older patients, and technical issues can occur with neuronavigation equipment, having additional superficial methods of estimating the underlying dural venous sinuses can be extremely useful.

Declarations

The authors sincerely thank those who donated their bodies to science so that anatomical research could be performed. Results from such research can potentially increase mankind’s overall knowledge which can then improve patient care. Therefore, these donors and their families deserve our highest gratitude [22].

## Conclusions

Although neuronavigation systems are widely adopted to identify deep intracranial structures, particularly dural venous sinuses (TS and TSJ) in posterior fossa surgery, additional reliable superficial anatomical landmarks, such as the OM, can be useful when this technology is not available or is not working properly.
